# Supplementary material for: ApoE4 associated with severe COVID-19 outcomes via downregulation of ACE2 and imbalanced RAS pathway
Source: J Transl Med. 2023 Feb 9;21:103. doi: 10.1186/s12967-023-03945-7 (PMC9910247; doi:10.1186/s12967-023-03945-7)
Supplement: Supplementary file 1 — Additional file 1: Fig. S1. Expression of ApoE and ACE2 in vitro. Representative western blotting analysis of the ApoE and ACE2 protein levels in A549, HEK-293T, SH-SY5Y, and HUVECs; the data are shown as the mean ± SD of three independent experiments. α-Tubulin was used as a loading control. P values were calculated using one-way ANOVA, *p < 0.05; **p < 0.01; ***p <0.001. Fig. S2. Molecular docking and simulation analyses of the interaction between ApoE and ACE2. A Molecular docking simulation of the SARS-CoV-2 RBD interacting with ACE2 (the orange region of the ACE2 protein represents the region that binds to the spike S1 protein). B and C Plot of backbone RMSD versus time (ns) for ApoE and ACE2. D Plot of Rg versus time (ns) for ApoE-ACE2. E Plot of total SASA versus time (ns) for ApoE-ACE2 complexes. F Number of hydrogen bonds between ApoE and ACE2. G Number of hydrophobic interactions between ApoE and ACE2. H Interaction energy between ACE2 and ApoE. Brown: ApoE2-ACE2, Green: ApoE3-ACE2 and Blue: ApoE4-ACE2. Fig. S3. ApoE4 downregulates ACE2 protein expression in vivo. ACE2 protein levels in the cortex, hippocampus, liver, bowel, spleen, kidney, heart and lung of ApoE2-TR, ApoE3-TR, and ApoE4-TR mice were assessed by immunofluorescence staining. The results were normalized to the expression of a-tubulin. n = 6 mice per group. The sections were stained with an anti-ACE2 (green) antibody and counterstained with DAPI (blue). The data are expressed as the mean ± SD. Statistical differences were evaluated by one-way ANOVA. Scale bars, 100 μm. *p < 0.05; **p < 0.01; ***p <0.001. Fig. S4. ApoE4 regulates Ang II and Ang 1-7 protein expression in vitro. Expression of the Ang II A and Ang 1-7 B proteins in HEK-293T cells as shown by ELISA after transfection with 1 µg/ml Flag, ApoE2-Flag, ApoE3-Flag or ApoE4-Flag plasmids for 48 h. The data are expressed as the mean ± SD. One-way ANOVA tests were used. *p<0.05. Table S1. Characteristics of the included studies. Table S2. [file 12967_2023_3945_MOESM1_ESM.doc]

**Additional file**

**
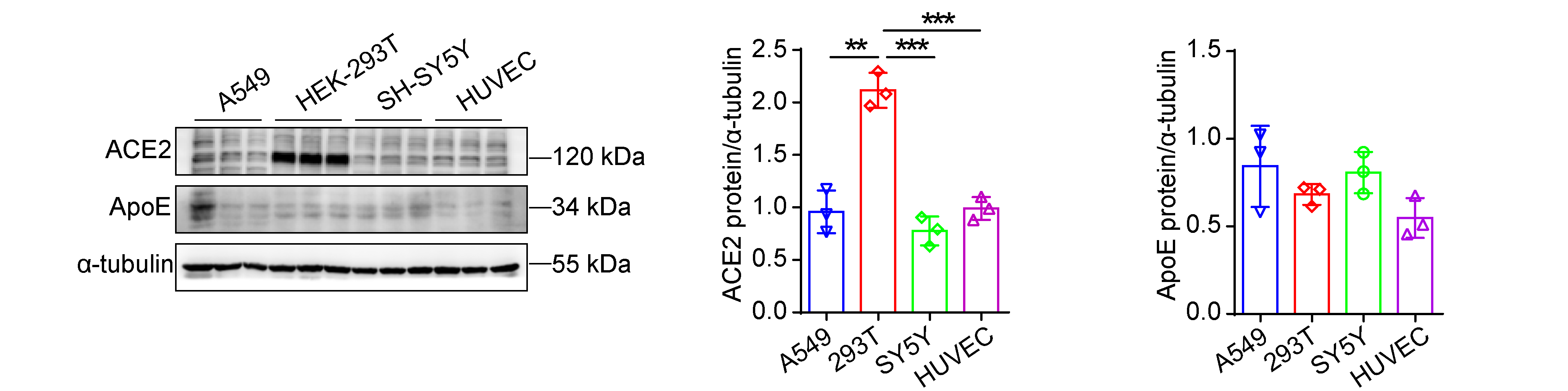
**

**Fig. S1** Expression of ApoE and ACE2 *in vitro*. Representative western blotting analysis of the ApoE and ACE2 protein levels in A549, HEK-293T, SH-SY5Y, and HUVECs; the data are shown as the mean ± SD of three independent experiments. α-Tubulin was used as a loading control. *P* values were calculated using one-way ANOVA, **p* < 0.05; ***p* < 0.01; ****p* <0.001.


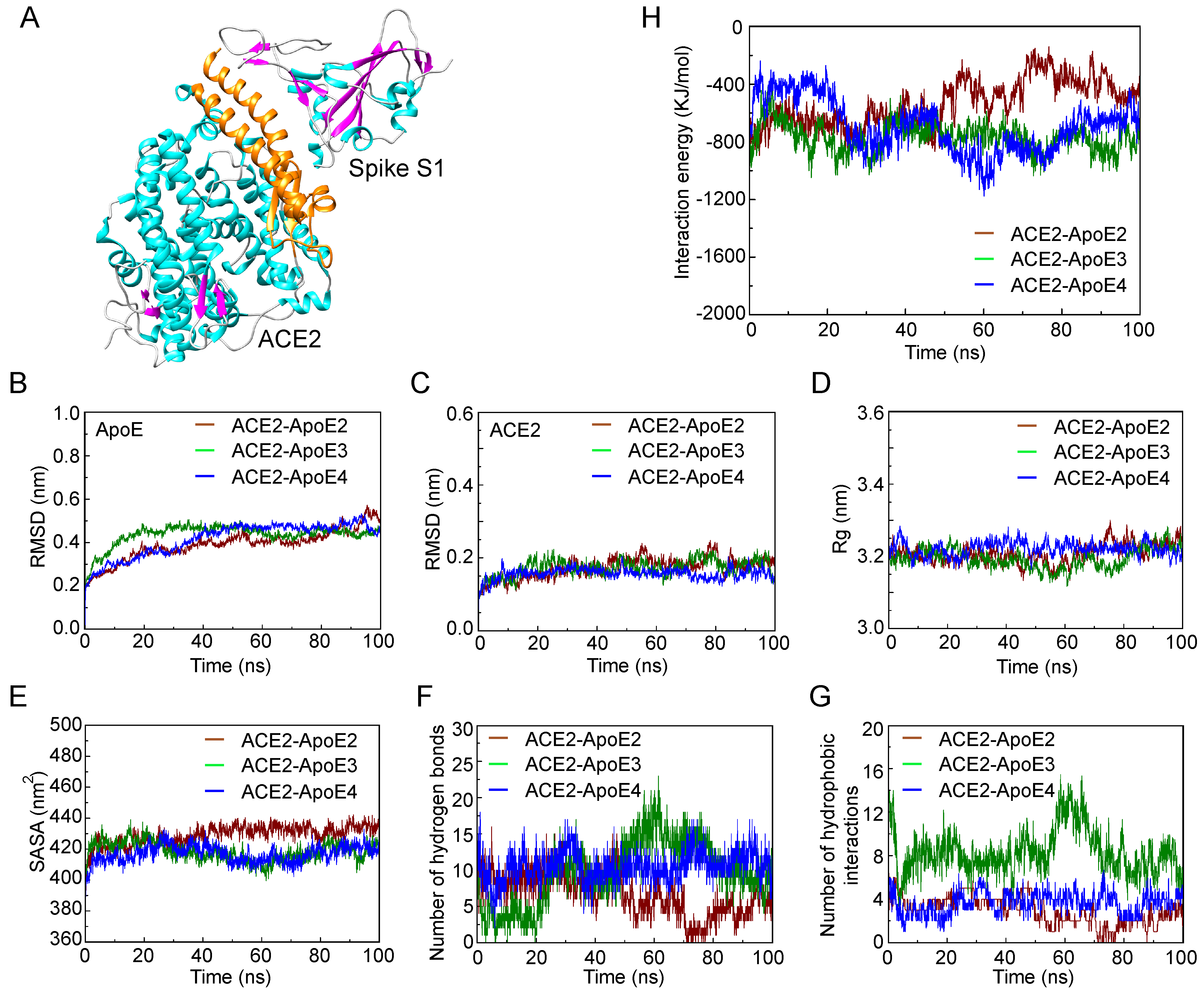


**Fig. S2** Molecular docking and simulation analyses of the interaction between ApoE and ACE2. **A** Molecular docking simulation of the SARS-CoV-2 RBD interacting with ACE2 (the orange region of the ACE2 protein represents the region that binds to the spike S1 protein). **B** and **C** Plot of backbone RMSD versus time (ns) for ApoE and ACE2. **D** Plot of Rg versus time (ns) for ApoE-ACE2. **E** Plot of total SASA versus time (ns) for ApoE-ACE2 complexes. **F** Number of hydrogen bonds between ApoE and ACE2. **G** Number of hydrophobic interactions between ApoE and ACE2. **H** Interaction energy between ACE2 and ApoE. Brown: ApoE2-ACE2, Green: ApoE3-ACE2 and Blue: ApoE4-ACE2.

**
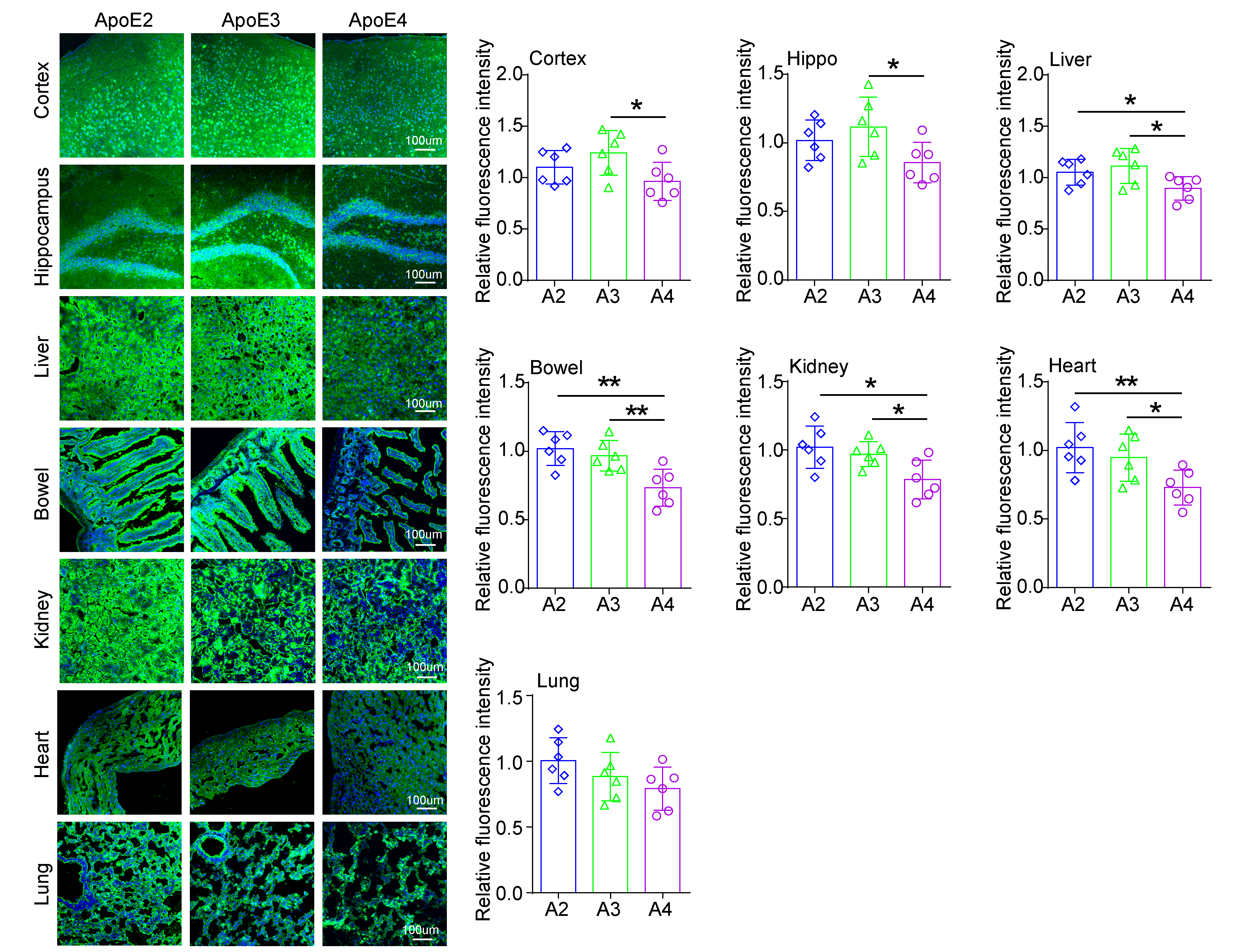
**

**Fig. S3** ApoE4 downregulates ACE2 protein expression *in vivo*. ACE2 protein levels in the cortex, hippocampus, liver, bowel, spleen, kidney, heart and lung of ApoE2-TR, ApoE3-TR, and ApoE4-TR mice were assessed by immunofluorescence staining. The results were normalized to the expression of a-tubulin. n = 6 mice per group. The sections were stained with an anti-ACE2 (green) antibody and counterstained with DAPI (blue). The data are expressed as the mean ± SD. Statistical differences were evaluated by one-way ANOVA. Scale bars, 100 μm. **p* < 0.05; ***p* < 0.01; ****p* <0.001.


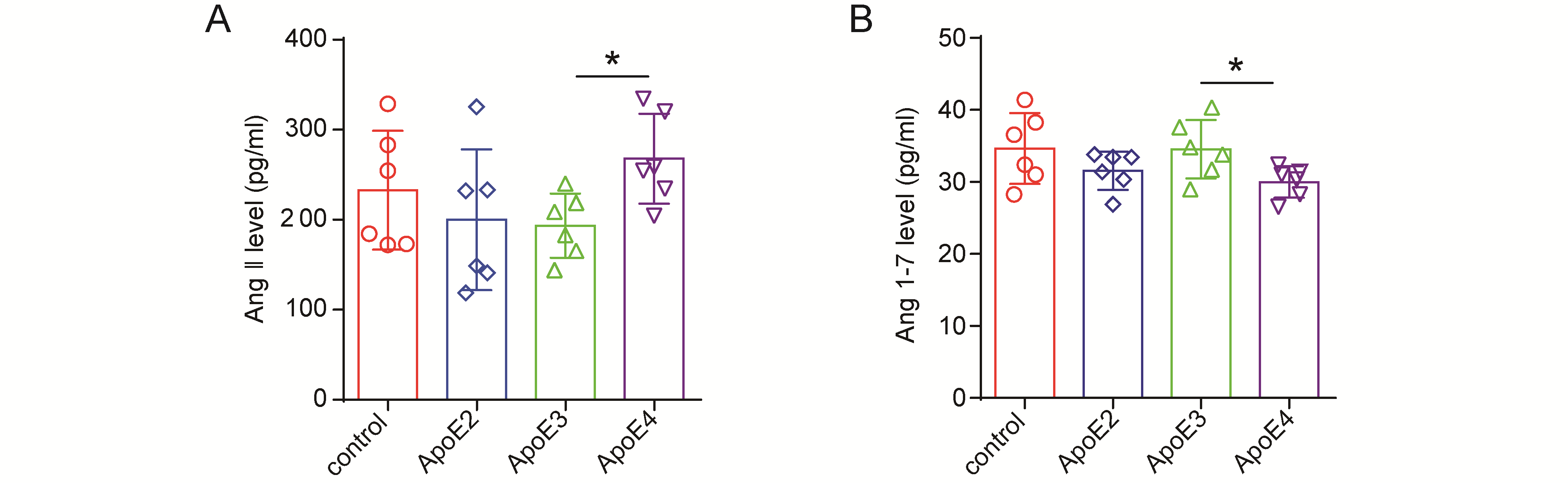


**Fig. S4** ApoE4 regulates Ang II and Ang 1-7 protein expression in vitro. Expression of the Ang II **A** and Ang 1-7 **B** proteins in HEK-293T cells as shown by ELISA after transfection with 1 µg/ml Flag, ApoE2-Flag, ApoE3-Flag or ApoE4-Flag plasmids for 48 h. The data are expressed as the mean ± SD. One-way ANOVA tests were used. **p*<0.05.

**Table S1. Characteristics of the included studies.**

| Study (author, reference) | Ethnity | Gender (M/F) |  | Genotypes | | | | | | Phenotype |
| --- | --- | --- | --- | --- | --- | --- | --- | --- | --- | --- |
|  |  |  |  | ε2/ε2 | ε2/ε3 | ε2/ε4 | ε3/ε3 | ε3/ε4 | ε4/ε4 |  |
| Al-Jaf, 2021 (28) | Iraq | 48/57 | control | 0 | 14 | 0 | 92 | 8 | 0 | risk |
|  |  |  | case | 0 | 10 | 3 | 77 | 15 | 0 |  |
| Kuo, 2020 (17) | UK | 14997/  176951 | control | - | - | - | 223056 | 90285 | 8985 | risk |
|  |  |  | case | - | - | - | 401 | 184 | 37 |  |
| Lord, 2022 (29) | Farmanfar-mayan | 122/79 | control | 2 | 7 | 2 | 83 | 7 | 0 | risk |
|  |  |  | case | 1 | 10 | 1 | 61 | 13 | 14 |  |
| Kuo, 2020 (18) | England | 142909/  174667 | control | - | - | - | 219084 | 88561 | 8708 | risk |
|  |  |  | case | - | - | - | 663 | 321 | 59 |  |
| Kurki, 2021 (13) | Finns | 1021/  1590 | control | APOE4+ (APOE4/E4 + APOE4/E3 + APOE4/E2) = 832 | | | | | | risk |
|  |  |  | Case in ICU | APOE4+ (APOE4/E4 + APOE4/E3 + APOE4/E2) = 24 | | | | | |  |
| Hubacek, 2021 (19) | Czech | 185/223 | control | APOE4+ (APOE4/E4 + APOE4/E3 + APOE4/E2) = 475 | | | | | | risk |
|  |  |  | case | APOE4+ (APOE4/E4 + APOE4/E3 + APOE4/E2) = 88 | | | | | |  |
| Hubacek, 2021 (19) | Czech | 185/223 | asymptomatic | APOE4+ (APOE4/E4 + APOE4/E3 + APOE4/E2) = 29 | | | | | | severity |
|  |  |  | symptomatic | APOE4+ (APOE4/E4 + APOE4/E3 + APOE4/E2) = 88 | | | | | |  |
| Kuo, 2020 (18) | England | 142909/  174667 | case | - | - | - | 219084 | 88561 | 8708 | severity |
|  |  |  | case with dead | - | - | - | 79 | 42 | 13 |  |
| del Ser, 2021 (30) | Spain | 323/590 | asymptomatic | APOE4+ (APOE4/E4 + APOE4/E3 + APOE4/E2) = 140 | | | | | | severity |
|  |  |  | symptomatic | APOE4+ (APOE4/E4 + APOE4/E3 + APOE4/E2) = 20 | | | | | |  |
| Kuo, 2022 (31) | England | 3595/  3830 | case | - | - | - | 941 | 358 | 64 | severity |
|  |  |  | Case with delirium | - | - | - | 52 | 43 | 13 |  |
| Tavares-Júnior, 2022 (32) | Brazil | 52/89 | control | 0 | 2 | 0 | 37 | 8 | 1 | severity |
|  |  |  | Case with cognitive impairment | 0 | 2 | 0 | 16 | 6 | 1 |  |

**Table S2. Newcastle-Ottawa Scale to assess quality of the involved studies.**

| Study  (first author, year, reference) | Selection | | | | Comparability | | outcome | | | |  |
| --- | --- | --- | --- | --- | --- | --- | --- | --- | --- | --- | --- |
|  | Adequate  case definition | Representa-  tiveness of the cases | Selection  of control | Definition  of control | | Control of  important  confusion factors | | Ascertainment  of exposure | Same method of  ascertainment for  cases and controls | Non-Response rate | Total score |
| Al-Jaf, 2021 (28) | ☆ | ☆ | ☆ | ☆ | | － | | ☆ | ☆ | ☆ | 7 |
| Kuo, 2020 (17) | ☆ | ☆ | ☆ | ☆ | | ☆ | | ☆ | ☆ | ☆ | 8 |
| Lord, 2022 (29) | ☆ | ☆ | ☆ | ☆ | | ☆ | | ☆ | ☆ | ☆ | 8 |
| Hubacek, 2021 (19) | ☆ | ☆ | ☆ | ☆ | | － | | ☆ | ☆ | ☆ | 7 |
| Kuo 2020 (18) | ☆ | ☆ | ☆ | ☆ | | ☆ | | ☆ | ☆ | ☆ | 8 |
| del Ser 2021 (30) | ☆ | ☆ | ☆ | ☆ | | ☆ | | ☆ | ☆ | ☆ | 8 |
| Kuo 2022 (31) | ☆ | ☆ | ☆ | ☆ | | ☆ | | ☆ | ☆ | ☆ | 8 |
| Kurki 2021 (13) | ☆ | ☆ | ☆ | ☆ | | ☆ | | ☆ | ☆ | ☆ | 8 |
| Tavares-Júnior 2022 (32) | ☆ | ☆ | ☆ | ☆ | | － | | ☆ | ☆ | ☆ | 7 |

☆ Represents one point.

**Table S3. Kinetic parameters of ApoE and ACE2 calculated by BLI.**

| Sample | Loading Sample | Concentration (nM) | Response | KD (M) | Kon (1 Ms) | Kdis (1/s) | Full R^2 |
| --- | --- | --- | --- | --- | --- | --- | --- |
| ApoE2 | ACE2 | 5882 | 0.0914 | 6.73E-07 | 3.42E+03 | 2.30E-03 | 0.93 |
|  |  | 2941 | 0.0503 |  |  |  |  |
|  |  | 1471 | 0.0353 |  |  |  |  |
|  |  | 735.3 | 0.0242 |  |  |  |  |
| ApoE3 | ACE2 | 5882 | 0.0896 | 7.08E-07 | 6.42E+03 | 4.54E-03 | 0.92 |
|  |  | 2941 | 0.0614 |  |  |  |  |
|  |  | 1471 | 0.0436 |  |  |  |  |
|  |  | 735.3 | 0.0268 |  |  |  |  |
| ApoE4 | ACE2 | 5882 | 0.1058 | 1.07E-06 | 2.85E+03 | 3.05E-03 | 0.93 |
|  |  | 2941 | 0.064 |  |  |  |  |
|  |  | 1471 | 0.0421 |  |  |  |  |
|  |  | 735.3 | 0.0303 |  |  |  |  |

**Table S4. Kinetic parameters of ApoE and SARS-CoV-2 (RBD) calculated by BLI.**

| Sample | Loading Sample | Concentration (nM) | Response | KD (M) | Kon (1 Ms) | Kdis (1/s) | Full R^2 |
| --- | --- | --- | --- | --- | --- | --- | --- |
| ApoE2 | SARS-Cov-2 | 5882 | 0.1713 | 1.71E-06 | 1.00E+04 | 1.72E-02 | 0.93 |
|  |  | 2941 | 0.1229 |  |  |  |  |
|  |  | 1471 | 0.0905 |  |  |  |  |
|  |  | 735.3 | 0.0637 |  |  |  |  |
|  |  | 367.6 | 0.0606 |  |  |  |  |
| ApoE3 | SARS-Cov-2 | 5882 | 0.1679 | 5.89E-07 | 1.22E+04 | 7.18E-03 | 0.91 |
|  |  | 2941 | 0.1183 |  |  |  |  |
|  |  | 1471 | 0.0884 |  |  |  |  |
|  |  | 735.3 | 0.0569 |  |  |  |  |
|  |  | 367.6 | 0.0562 |  |  |  |  |
| ApoE4 | SARS-Cov-2 | 5882 | 0.1476 | 4.88E-07 | 12530 | 0.00611 | 0.89 |
|  |  | 2941 | 0.1074 |  |  |  |  |
|  |  | 1471 | 0.0779 |  |  |  |  |
|  |  | 735.3 | 0.0583 |  |  |  |  |
|  |  | 367.6 | 0.0539 |  |  |  |  |
